# Racial Disparities in the Epidemiology of COVID-19 in Georgia: Trends Since State-Wide Reopening

**DOI:** 10.1089/heq.2020.0089

**Published:** 2021-03-02

**Authors:** Grace Porter, Koosh Desai, Varghese George, Steven S. Coughlin, Justin Xavier Moore

**Affiliations:** ^1^ Institute of Preventive and Public Health, Medical College of Georgia, Augusta University, Augusta, Georgia, USA.; ^2^ Department of Medicine, Medical College of Georgia, Augusta University, Augusta, Georgia, USA.; ^3^ Department of Population Health Sciences, Augusta University, Augusta, Georgia, USA.

**Keywords:** COVID-19, novel coronavirus, SARS-CoV-2, Georgia, social determinants, geographic distribution

## Abstract

**Objective:** To examine county-level factors associated with coronavirus disease 2019 (COVID-19) incidence and mortality in Georgia, focusing on changes after relaxation of “shelter-in-place” orders on April 24, 2020.

**Methods:** County-level data on confirmed COVID-19 cases and deaths were obtained from the Johns Hopkins 2019 Novel Coronavirus Data Repository and linked with county-level data from the 2020 County Health Rankings. We examined associations of county-level factors with mortality and incidence rates (quantiles) using a logistic regression model. This research was conducted in June–July 2020 in Augusta, GA.

**Results:** Counties in the highest quartile for mortality had higher proportions of non-Hispanic (NH)-Black residents (median: 37.4%; interquartile range [IQR]: 29.5–45.0; *p*<0.01) and residents with incomes less than $20,000 (median: 32.9%; IQR: 26.6–35.0; *p*<0.01). Counties in the highest quartile for NH-Black residents (38.7–78.0% NH-Black population) showed a 13-fold increase in odds (odds ratio=13.15, 95% confidence interval=1.40–123.80, *p*=0.05) for increased COVID-19 mortality controlling for income.

**Conclusions:** Although highlighted by the pandemic, racial disparities predated COVID-19, exposing the urgency for diversion of resources to address the systematic residential segregation, educational gaps, and poverty levels experienced disproportionately by Black communities.

## Introduction

As part of an ongoing public health crisis, the novel coronavirus disease 2019 (COVID-19) continues to devastate the United States with over 4 million confirmed cases and more than 150,000 deaths as of July 30, 2020.^1^ Although COVID-19 exerts an effect on all U.S. citizens in some capacity, minority populations, particularly non-Hispanic (NH) Blacks, are disproportionately affected in incidence and mortality of COVID-19. Counties with a higher proportion of NH-Black residents, defined as greater than the national average (>13%), represented just 22% of the 3142 sampled counties across the United States, yet accounted for 52% of COVID-19 cases and 58% of COVID-19-related deaths even after adjustment for average age, poverty level, comorbidities, and epidemic exposure time.^2^

In New York City, one of the early epicenters of COVID-19, boroughs such as the Bronx with higher proportions of NH-Black residents exhibited the highest number of hospitalizations and deaths related to COVID-19. These hospitalizations and deaths occurred despite having lower population densities, younger overall population, and adequate hospital bed numbers compared to less diverse and more affluent boroughs such as Manhattan.^3^ Boroughs like the Bronx also exhibited a greater proportion of residents living in below the federal poverty line and with an overall lower level of education attainment, pointing to a multifactorial systematic exposure risk. In a large California health system, NH-Black patients with COVID-19 had a 2.7-fold increased risk of hospitalization from the disease compared to their NH-White counterparts, after adjustment for age, comorbidities, sex, and income.^4^ Disparities in COVID-19 incidence and mortality among racial minorities are complex and multifactorial, but may be explained, in part, by persistent poverty and structural inequities.^5,6^

In Georgia, COVID-19-related health disparities emerged in the first 7 weeks of community spread across the state, in terms of both race and availability of health care resources.^7^ Although urban areas such as Metropolitan Atlanta exhibited the largest number of confirmed cases, more rural southwestern counties such as those surrounding Albany, exhibited the highest biweekly increase in incidence and mortality rates per 100,000 population.^7^ In addition, counties with the highest rates of mortality in the first 7 weeks of community spread were also more likely to have a higher proportion of residents who were NH-Black, older than 60 years, living with yearly incomes less than $20,000, and living in rural areas. Theses counties were also more likely to have fewer intensive care unit (ICU) beds per 100,000 people and fewer primary care physicians per 10,000 people, indicating a possible deficiency in access to medical care and ability to handle outbreaks of pandemic scale.^7^

Despite increases in confirmed daily new case numbers, Georgia was one of the first states to ease “stay-at-home” policy on April 24, 2020, in an attempt to stave off further economic downturn initiated by social distancing guidelines. In this study, we examine the county-level trends in COVID-19 incidence and mortality on a monthly basis through June. Building upon our previous research that included data predating the relaxation of such restrictions,^7^ this study investigates the trends of elevated incidence and mortality rates of COVID-19 and examines differences by sociodemographic factors to assess if reopening the state exacerbated or alleviated such disparities.

Assessing differences in incidence and mortality will help to further quantify the health disparities previously seen in the COVID-19 outbreak. In addition, illustration of factors that may predispose an area to higher incidences and mortality rates can assist in providing targeted public health approaches that assess specific regional needs and risk factors. These data also have broader implications for future identification of especially vulnerable areas and populations that may have yet to experience COVID-19, but would benefit from prophylactic public health intervention to prevent subsequent morbidity and mortality.

## Methods

### Study design and setting

We examined the geographic variation of COVID-19 in the state of Georgia from March 3 through June 30, 2020, using county-level data. Since the first case was confirmed on March 2, the Georgia Department of Public Health (GADPH) has provided a daily update of confirmed cases of COVID-19, tested both by commercial laboratories as well as the state department using the CDC 2019-nCoV real-time reverse transcription-polymerase chain reaction (RT-PCR) diagnostic tests.^8^ Sensitivity has been estimated to range from 65% to 100% depending on RNA concentrations.^9^ We obtained the county-level data on COVID-19 confirmed cases and deaths from the Johns Hopkins 2019 Novel Coronavirus Data Repository,^10^ which imports the GADPH daily status report file for each county, allowing for investigators to examine the trend and aggregate number of cases and deaths. We linked the county-level COVID-19 data with the county-level data on sociodemographic, access to health care, and hospital critical care infrastructure factors, derived from the 2020 County Health Rankings, 2014 American Community Survey, 2010 Census, and 2017–2019 Centers for Medicare & Medicaid Services hospital reports. This study was considered exempt by Institutional Review Board review because we used publicly available, de-identified secondary data.

### Statistical analysis

**FIG. 1.**
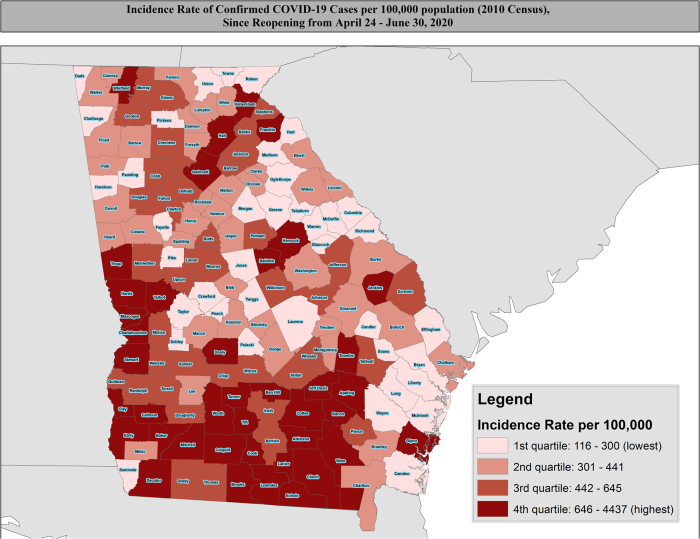

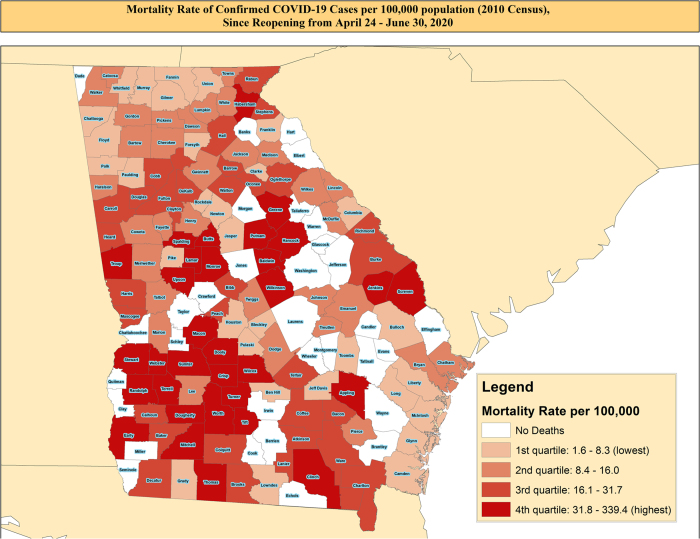
**(A)** Geographic distribution of incidence rates of COVID-19 pandemic in Georgia counties since April 24th presented as per 100,000 population, based on 2010 U.S. Census Estimates for counties. **(B)** Geographic distribution of mortality rates of COVID-19 pandemic in Georgia counties since April 24th presented as per 100,000 population, based on 2010 U.S. Census Estimates for counties. COVID-19, coronavirus disease 2019.

We calculated four measures of disease morbidity at the county level: (1) the monthly confirmed case incidence rate per 100,000 population from March through June (Presented in Table 1); (2) monthly confirmed case mortality rate per 100,000 population from March through June (Presented in Table 1); (3) the overall incidence rate per 100,000 population since reopening on April 24th through June 30th; and (4) the overall mortality rate per 100,000 population since reopening on April 24th through June 30th. These results are presented in Figures 1A and B. We compared the county-level characteristics of the population such as demographic factors by mortality rate quintiles and incidence rate quartiles using Kruskal–Wallis tests, and the results are given in Tables 2 and 3 as well as in Supplementary Tables S1 and S2. In the secondary analysis, we examined county-level differences in incidence and mortality rates, comparing (1) counties with <13% NH-Black residents versus those with ≥13%; (2) counties with <9% Hispanic residents versus those with ≥9%; and (3) counties with <20% of residents having yearly incomes less than $20,000 versus those with ≥20% of residents with yearly incomes less than $20,000. These results are presented in Table 1. We performed all statistical analysis using SAS version 9.4 and executed all mapping using ArcGIS version 10.5.

**Table 1.** Incidence and Mortality Rates Over First 4 Months of COVID-19 Pandemic in Georgia

	March	April	May	June
	Presented as rate per 100,000 persons (95% CI)			
Incidence				
>13% NH-Black population	41.1 (39.72–42.52)	215.95 (212.76–219.19)	175.62 (172.75–178.55)	323.17 (319.26–327.12)
<13% NH-Black population	27.76 (25.33–30.43)	207.77 (200.92–214.85)	209.96 (203.07–217.08)	263.91 (256.17–271.87)
>9% Hispanic population	36.20 (34.47–38.02)	210.25 (206.01–214.58)	222.77 (218.40–227.22)	354.89 (349.37–360.50)
<9% Hispanic population	41.02 (39.33–42.79)	218.15 (214.21–222.17)	147.08 (143.84–150.38)	278.32 (273.86–282.85)
>20% Living with less than $20 K	37.15 (35.29–39.10)	232.35 (227.63–237.16)	173.71 (183.26–190.32)	358.11 (352.25–364.07)
<20% Living with less than $20 K	39.99 (38.39–41.66)	202.39 (198.75–206.10)	186.76 (183.26–190.33)	282.30 (277.99–286.67)
Mortality				
>13% NH-Black population	1.23 (1.01–1.50)	10.87 (10.17–11.61)	9.55 (8.90–10.25)	7.49 (6.91–8.11)
<13% NH-Black population	0.67 (0.37–1.21)	6.50 (5.38–7.86)	5.77 (4.72–7.06)	5.77 (4.72–7.06)
>9% Hispanic population	0.93 (0.69–1.27)	7.95 (7.16–8.83)	8.79 (7.96–9.72)	8.41 (7.20–9.82)
<9% Hispanic population	1.31 (1.03–1.65)	11.93 (11.04–12.90)	9.00 (8.23–9.85)	6.90 (6.34–7.51)
>20% Living with less than $20 K	1.22 (0.92–1.62)	13.82 (12.71–15.03)	9.57 (8.66–10.60)	8.74 (7.86–9.71)
<20% Living with less than $20 L	1.08 (0.84–1.38)	7.60 (6.92–8.34)	8.45 (7.73–9.24)	6.14 (5.53–6.81)

Rates presented per 100,000 population, based on 2010 U.S. Census Estimates.

CI, confidence interval; NH, non-Hispanic.

**Table 2.** County-Level Characteristics Comparison by COVID-19 Mortality Rates per 100,000, Among Georgia Counties Since Reopening on April 24, 2020

	Quartiles of mortality rate					
Characteristic	Counties with no deaths (n=32)	1st Quartile (1.6–8.4) (n=31)	2nd Quartile (8.4–16.4) (n=32)	3rd Quartile (16.4–32.8) (n=32)	4th Quartile (32.8–339.4) (n=32)	p^a^
	Presented as median (IQR)					
Race						
% NH-White	61.8 (54.3–75.7)	71.1 (58.4–82.6)	77.6 (55.4–93.1)	56.3 (43.6–75.7)	53.3 (40.2–63.8)	<0.0001
% NH-Black	25.3 (18.8–35.3)	23.1 (9.5–33.6)	21.7 (4.6–33.6)	29.7 (16.6–46.3)	37.4 (29.5–45.0)	0.0001
% Hispanic	4.0 (2.5–6.5)	7.5 (4.5–12.2)	4.7 (3.0–8.0)	6.9 (5.0–11.6)	3.9 (2.5–5.8)	0.0002
% Female sex	49.0 (47.3–51.1)	53.0 (51.2–57.3)	53.2 (50.4–56.7)	51.6 (49.4–56.6)	49.0 (47.2–52.2)	<0.0001
% Age 65+	17.6 (16.6–20.1)	16.2 (14.2–18.0)	18.1 (16.3–20.1)	15.5 (14.1–17.5)	17.8 (15.9–18.5)	0.0040
ICU^b^ beds per 100,000 population	0.0 (0.0–0.0)	16.0 (0.0–30.1)	0.0 (0.0–14.7)	8.5 (0.0–28.2)	0.0 (0.0–28.0)	0.0011
PCP per 10,000 population	3.0 (1.5–4.5)	4.6 (2.7–6.2)	4.5 (2.8–5.8)	4.1 (2.4–6.8)	3.7 (1.4–5.6)	0.1081
% Uninsured	12.4 (11.1–13.8)	14.0 (12.3–15.2)	12.5 (12.0–14.5)	13.5 (11.7–15.4)	12.0 (11.1–13.0)	0.0041
% Income <$20,000	29.2 (23.9–33.4)	24.1 (19.1–28.9)	22.4 (16.2–29.4)	25.5 (17.8–31.5)	32.9 (26.6–35.0)	<0.0001
% Attained some college education	11.4 (9.3–12.5)	12.1 (10.3–18.0)	12.7 (10.3–16.4)	12.8 (10.5–16.8)	10.4 (9.4–12.2)	0.0047
% Adult obesity	26.4 (22.2–29.8)	26.2 (23.8–29.0)	24.0 (22.6–27.3)	26.1 (23.6–27.4)	25.8 (24.0–28.3)	0.3089
% Adult smoking	18.8 (17.8–20.2)	17.8 (16.4–19.1)	17.2 (15.7–18.3)	18.3 (17.0–19.8)	19.7 (18.1–21.3)	0.0001
% Rural	75.0 (67.1–100.0)	51.1 (23.2–74.3)	66.2 (35.7–83.4)	50.7 (18.2–73.1)	59.6 (47.0–78.4)	<0.0001

^a^ Significance determined using Kruskal–Wallis tests, *p*<0.05.

^b^ ICU bed tally does not include Veterans Affairs hospitals, which are sure to play a role in treating COVID-19 patients, because VA hospitals do not file cost reports to CMS.

CMS, centers for medicare & medicaid Services; COVID-19, coronavirus disease 2019; ICU, intensive care unit; IQR, interquartile range; PCP, primary care physicians.

**Table 3.** The Association Between County-Level Characteristics with Highest Quartile of COVID-19 Mortality Rates, Among Georgia Counties Since Reopening on April 24, 2020

Characteristic	Unadjusted OR^a^ (95% CI)	p^b^	Adjusted^c^ OR (95% CI)	p^b^	Adjusted^d^ OR (95% CI)	p^b^
% NH-Black						
1st quartile (0.7–14.7)	1.00 (Referent)	0.014	1.00 (Referent)	0.13	1.00 (Referent)	0.05
2nd quartile (14.8–28.0)	6.71 (0.77–58.55)	0.990	7.67 (0.77–70.99)	0.579	9.03 (0.74–110.90)	0.896
3rd quartile (28.1–38.7)	14.41 (1.76–118.10)	0.0494	10.99 (1.26–96.10)	0.107	17.72 (1.55–202.90)	0.118
4th quartile (38.7–78.0)	20.46 (2.53–165.29)	0.003	13.15 (1.40–123.80)	0.05	31.46 (2.43–408.00)	0.013
% Hispanic						
1st quartile (1.4–3.1)	1.00 (Referent)	0.056	1.00 (Referent)	0.279	1.00 (Referent)	0.429
2nd quartile (3.2–5.1)	0.85 (0.32–2.26)	0.166	1.09 (0.83–3.10)	0.166	1.53 (0.46–5.04)	0.126
3rd quartile (5.2–8.5)	0.32 (0.10–1.02)	0.247	0.48 (0.14–1.64)	0.434	0.60 (0.15–2.40)	0.481
4th quartile (8.6–36.4)	0.25 (0.07–0.86)	0.097	0.39 (0.10–1.39	0.208	0.52 (0.11–2.43)	0.371
% Income <$20,000						
1st quartile (8.7–19.4)	1.00 (Referent)	0.001	1.00 (Referent)	0.05	1.00 (Referent)	0.05
2nd quartile (19.5–27.0)	3.92 (0.76–20.23)	0.844	4.82 (0.84–27.55)	0.308	2.58 (0.35–18.96)	0.292
3rd quartile (27.1–32.1)	3.27 (0.62–17.28)	0.787	2.56 (0.46–14.30)	0.633	0.77 (0.10–5.99)	0.161
4th quartile (32.2–47.8)	13.67 (2.89–64.74)	<0.0001	7.67 (1.52–38.67)	0.014	2.87 (0.38–21.57)	0.185

Presented as ORs and 95% CIs, estimated using binary logistic regression.

^a^ OR estimate the odds of being in the highest quartile for COVID-19 mortality rate.

^b^ Significance determined using Wald Chi-square from logistic regression.

^c^ Adjusted for the reciprocal (income or race) variable.

^d^ Adjusted form the reciprocal (income or NH-Black proportion) variable and, age, obesity, and rurality.

ORs, Odds ratios.

## Results

### Geographic distribution of incidence and mortality across Georgia

Since the easing of “shelter-in-place” restrictions on April 24th, higher incidence rates rapidly expanded to more counties across the state from the original epicenter in southwest Georgia (Fig. 1A). In addition, southwest Georgia remained a hotspot for high mortality seen in the first 7 weeks,^7^ but more areas like central and eastern Georgia experienced increasing mortality through May and June (Fig. 1B). The month-by-month distributions of county-level incidence and mortality for COVID-19 per 100,000 population are presented in Supplementary Figures S1 and S2, respectively.

### Racial and income disparities for incidence and mortality

Overall, incidence rates of COVID-19 generally increased, while mortality rates stagnated across all counties in Georgia over the 4-month period since the first infection (Table 1). However, racial and income disparities emerged early and persistently for incidence and mortality. Earlier in the pandemic during the months of March and April, counties with less than 9% Hispanic population had higher incidence of COVID-19 compared with counties with greater than 9% Hispanic population. However, after reopening and during the months of May and June, counties with greater than 9% Hispanic population had higher incidence rates when compared to counties with lower than 9% Hispanic population. Monthly mortality rates between counties with differing Hispanic populations were not as disparate. In counties with NH-Black population greater than 13%, incidence rates were initially slightly higher than in counties with less than 13% NH-Black residents early in the pandemic course, reaching a peak in April. After May, however, the incidence rates in counties with greater than 13% NH-Black residents were greater than counties with less than 13% NH-Black residents. The mortality rates for counties with greater than 13% NH-Black residents remained greater compared with counties with fewer NH-Black residents during all 4 months, indicating a consistent pattern of disparity in disease burden among counties with greater NH-Black population. Graphical representations of these patterns are shown in Supplementary Figure S3A.

To understand the differences in COVID-19 incidence mortality by county-level poverty, we compared counties with greater than 20% of residents with annual income less than $20,000 to those counties with less than 20% of residents earning annual income less than $20,000 (Table 1). For counties having greater than 20% of residents making less than $20,000 annually, the monthly incidence rates were greater compared with wealthier counties with the exception of the month of May. In June, less affluent counties begin to see increasing incidence rates greater compared with wealthier counties. In addition, counties with more than 20% of residents making less than $20,000 a year had a higher mortality rate across all 4 months, indicating that counties that experience more poverty also experience a greater burden of disease, as shown graphically in Supplementary Figure S3B**.**

### County-level characteristics and their association with incidence and mortality rates

Counties within the highest quartile for COVID-19 mortality had higher proportions of NH-Black residents (median: 37.4%; interquartile range [IQR]: 29.5–45.0; *p*<0.01), fewer female residents (median: 49.0%; IQR: 47.2–52.2; *p*<0.01), more residents with income less than $20 K (median: 32.9%; IQR: 26.6–35.0; *p*<0.01), higher adult smoking prevalence (median: 19.7%; IQR: 18.1–21.3; *p*<0.01), and less overall college education (median: 10.4%; IQR: 9.4–12.2; *p*<0.01) (Table 2).

Counties in the highest quartile for NH-Black residents (32.2–47.8% NH-Black population) had 20-fold increased odds (odds ratio [OR]=20.65, 95% confidence interval [CI]=2.53–165.29, *p*=0.003) of being in the highest quartile for COVID-19 mortality (Table 3). Counties in the highest quartile for NH-Black residents (38.7–78.0%) showed a 13-fold increase in odds (OR=13.15, 95% CI=1.40–123.80, *p*=0.05) for increased mortality after adjustments for potential income confounders, although the overall model was not significant. Increased odds of being in the highest quartile for COVID-19 mortality were not observed for counties with the highest quartile (8.6–36.4%) of Hispanic population in the unadjusted or adjusted models, and the models were not statistically significant. Similarly, counties within the highest quartile for poverty level (containing the highest proportion of residents living with <$20 K per year) had a 13-fold increase in likelihood (OR=13.67, 95% CI=2.29–64.74, *p*<0.01) of being in the highest quartile for mortality (Table 3). Counties in the highest quartile for increased poverty showed a 7-fold increase in odds (OR=7.67, 95% CI=1.52–38.67, *p*=0.014) for increased mortality after adjustments for potential racial confounders (Table 3).

In a multivariable analysis controlled for obesity rates, degree of rurality, and proportion of population older than 65 years, the likelihood of attaining higher mortality for counties with the largest NH-Black populations increased (adjusted OR=31.46, 95% CI=2.43–408.00, *p*=0.013), while the odds of attaining higher mortality for counties with high levels of poverty became nonsignificant (adjusted OR=2.87, 95% CI=0.38–21.57, *p*=0.135), indicating interplay between poverty and other confounding factors in the model (Table 3).

Since the relaxation of stay-at-home orders on April 24th, counties within the highest quartile of COVID-19 incidence had a lower proportion of NH-White population (median: 56.0%; IQR: 42.8–63.9; *p*=0.0004), lower income (median: 31.7%; IQR: 25.7–35.1; *p*=0.002), and higher adult smoking prevalence (median: 19.4%; IQR: 18.2–20.5; *p*=0.0008) (Supplementary Table S2).

When examining the disparity between NH-Black versus other residents in COVID-19 incidence using the data from March through June, we found no significant association (Supplementary Table S3). However, counties with the highest proportion of residents earning less than $20,000 annual had 6-fold increased odds of being in the highest quartile of COVID-19 incidence (4th vs. 1st quartile OR=6.47, 95% CI=1.93–21.67, *p*=0.003). After further adjustments for obesity rates, degree of rurality, and proportion of population older than 65 years, counties with more residents earning less than $20,000 annually had an18-fold increased odds (OR=18.24, 95% CI=3.40–97.92, *p*=0.002) of being in the highest quartile for incidence.

The trends observed since the easing off “shelter-in-place” orders on April 24th are similar to those observed since the first documented case in Georgia, illustrating that racial and income disparities remain as the pandemic continues (Supplementary Tables S3).

## Discussion

The racial disparities identified in the first 7 weeks of the pandemic^7^ persisted in subsequent months and after state-wide reopening. Counties with >13% NH-Black residents had markedly higher monthly mortality rates despite fluctuations in the incidence compared to counties with <13% NH-Black residents. Similar trend is also observed when 50% cutoff is used instead of 13%. In addition, counties in the highest quartile mortality since reopening tended to display a greater proportion of NH-Black residents, male residents, undereducated, tobacco users, and residents with lower poverty level compared to those in the lower mortality quartiles. For counties with the highest proportion of NH-Black residents, the odds of experiencing higher mortality were 20-fold greater compared to less diverse counties. While racial disparities in incidence rates between counties were not observed to the same degree, the consistently higher mortality rates indicate that counties with larger proportions of NH-Black residents are experiencing worse outcomes without an accompanying parallel increase in community spread.

Physiological theories for disparate outcomes in COVID-19 have been suggested, notably a differential cytokine expression among races, with a larger proinflammatory immune response during COVID-19 infection and elevated IL-6 levels in NH-Black individuals compared to their White counterparts.^11^ Structural inequities and systemic biases that predated the pandemic have also gained traction as an explanation for the stark differences in COVID-19 incidence and mortality. In Chicago, along with an increased number of COVID cases and resulting deaths, regions with majority NH-Black residents also exhibited higher social vulnerability scores indicating disadvantages in education, employment, and income level. In addition, many of these counties exhibited higher health risk factor scores, demonstrating a higher risk of comorbidities such as asthma, obesity, stroke, and cardiac-related death.^12^ The health and wealth inequities experienced by the African American community put them at the greatest risk for COVID-19 infection and mortality.

Counties with greater than 20% of residents with an income less than $20,000 had a greater monthly incidence and mortality than counties with less than 20% of residents in the income group. Individuals with lower incomes are more likely to hold state-determined “essential jobs”^6^ such as food service, grocery stores, sanitation workers, and retail/in-store customer service. These workers are thus placed at an increased risk of exposure to COVID-19 through common contact with the general population, when compared to someone of higher socioeconomic status, which may work in a standalone office for instance. In fact, while the correlation between percentage of COVID-19 deaths and all occupations was positive, the correlation was much higher^13^ for essential professions such as food preparation and transport services, which cannot translate to remote working modalities.

The increased exposure from working essential jobs could contribute to the finding that counties with a greater percentage of individuals experiencing poverty saw an unadjusted 6-fold increase in incidence. The difference in incidence between these income groups increased sharply in late May, possibly due to the impact of the economic strain^6^ of the pandemic on individuals of lower socioeconomic status, forcing a premature return to work despite ongoing public health concerns. Furthermore, regarding the racial demographics of essential professions, NH-Black employees were disproportionately represented in the top nine occupations, all deemed essential, which placed them in the highest risk for COVID-19 exposure and contractility compared to NH-White employees.^13^ The overrepresentation of NH-Black citizens working in essential jobs lends credence to the notion that the observed increases in odds of mortality can be more aptly attributed to systemic inequities rather than physiological differences between racial groups.

Counties with greater proportion of NH-Black residents, male residents, undereducated, poverty levels, tobacco users, and residents with lower poverty level retained higher incidence and mortality rates as noted in the early era of COVID-19. However, known risk factors such as increased age and obesity levels were not significantly associated with the highest incidence and mortality quartiles, as seen in earlier months. In addition, rurality and health resource scarcity were not associated with the COVID-19 as strongly as in the first 7 weeks of COVID-19,^7^ which could be a matter of how widespread the virus has become throughout Georgia. Also, as the number of cases and hospitalizations have begun to increase greatly in July, resource inequality may reemerge as an important factor, especially as southeast rural Georgia currently contends with a shortage of ICU beds.^14^ Although counties with low mortality tend to be more rural since reopening, they exhibit characteristics that make them especially vulnerable to high incidence and mortality from COVID-19.

### Limitations

The results of this study should be interpreted in light of a few limitations. As we performed an ecologic study at the county level, it is possible that COVID-19 cases were infected in counties that are different from where they died. Due to the diversity of COVID-19 infection presentations, underreporting of pre-symptomatic or asymptomatic cases could contribute to an underestimation of the true burden of disease. In addition, due to concerns about patient privacy and protected patient information, this study lacked individual patient-level data and estimation of racial disparities in disease burden was not feasible. More extensive studies examining individual-level racial disparities in mortality and outcome are needed to best assess and address the racial disparities seen on an ecological level.

## Conclusion

Given these results, state and local authorities can identify vulnerable counties and populations, which is important for just and appropriate resource allocation and support during the subsequent months of the pandemic. Effective identification can allow for effective response in the most affected areas to educate the public on the importance of reducing community spread through social distancing measures, while also highlighting populations who may be even more susceptible to disease to allow for enhanced precautions. Policy-level actions can be enacted to address the underlying inequities that preceded and continued during the pandemic to help protect vulnerable populations against future health disparities, together with enhanced public awareness of the inequities to help ignite changes at community and legislative levels.

For counties yet to experience high mortality, but which are especially vulnerable demographically, recognition and subsequent involvement from public health and health care officials will allow for prophylactic educational intervention on the importance of masks, social distancing, and other protective measures to mitigate the high incidence and mortality seen in similar counties. Although highlighted by the pandemic, racial disparities predated COVID-19, exposing the urgency of diversion of resources to address the systematic residential segregation, educational gaps, and poverty levels experienced disproportionately by the Black community.

## Supplementary Material

Supplemental data

Supplemental data

Supplemental data

Supplemental data

Supplemental data

Supplemental data

## Abbreviations Used

CI: confidence interval
CMS: centers for medicare & medicaid services
COVID-19: coronavirus disease 2019
GADPH: Georgia Department of Public Health
ICU: intensive care unit
IQR: interquartile range
NH: non-Hispanic
OR: odds ratio
PCP: primary care physicians
RT-PCR: reverse transcription-polymerase chain reaction
