# Supplementary material for: Racial Disparities in the Epidemiology of COVID-19 in Georgia: Trends Since State-Wide Reopening
Source: Health Equity. 2021 Mar 2;5(1):91–9. doi: 10.1089/heq.2020.0089 (PMC7990566; doi:10.1089/heq.2020.0089)
Supplement: Supplemental data [file Supp_Table1.docx]

| **Supplemental Table 1. County Level Characteristics Comparisons by Coronavirus Disease 2019 (COVID-19) Mortality Rates, Among Georgia Counties March 3 through on June 30, 2020.** | | | | | | |
| --- | --- | --- | --- | --- | --- | --- |
|  | Quartiles of Mortality Rate | | | | |  |
| Characteristic | Counties with No Deaths  (N = 19) | 1^st^ Quartile (1.6-11.4)  (*N* = 35) | 2^nd^ Quartile (11.4-22.7)  (*N* = 35) | 3^rd^ Quartile (22.7-43.0)  (*N* = 35) | 4^th^ Quartile (43.0-339.4)  (*N* = 35) | ***p* value^a^** |
| Presented as Median (IQR)^b^ | | | | | | |
| Race |  |  |  |  |  |  |
| % NH-White | 62.7 (57.4-76.2) | 69.8 (56.5-86.7) | 71.1 (59.1-92.3) | 56.5 (44.7-75.5) | 53.6 (35.5-64.1) | **<0.0001** |
| % NH-Black | 23.9 (18.2-28.6) | 25.9 (5.1-36.1) | 20.7 (8.0-31.7) | 30.2 (16.2-45.1) | 36.9 (27.0-47.4) | **0.0235** |
| % Hispanic | 5.6 (3.4-11.5) | 5.1 (3.1-7.7) | 6.5 (3.3-11.1) | 6.1 (3.4-11.0) | 4.2 (2.5-5.7) | **0.2276** |
| % Female Sex | 49.0 (47.4-50.8) | 51.7 (49.6-56.4) | 53.3 (51.2-57.3) | 51.8 (49.5-56.5) | 48.9 (45.6-52.2) | **0.0003** |
| % Age 65+ | 17.6 (15.6-20.4) | 16.7 (14.5-19.5) | 17.4 (16.1-18.7) | 15.5 (14.1-18.0) | 17.8 (15.7-19.0) | **0.1405** |
| ICU^c^ beds per 100,000 population | 0.0 (0.0-0.0) | 0.0 (0.0-21.1) | 0.0 (0.0-25.7) | 8.6 (0.0-24.3) | 0.0 (0.0-26.2) | **0.3050** |
| PCP^d^ per 10,000 population | 2.7 (1.3-4.0) | 3.6 (2.9-5.5) | 5.0 (2.6-7.2) | 4.4 (2.5-7.2) | 3.4 (1.5-5.6) | **0.5080** |
| % Uninsured | 13.0 (11.3-14.1) | 12.9 (11.8-15.2) | 13.0 (12.2-14.7) | 12.7 (11.7-15.5) | 12.1 (10.9-13.5) | **0.2569** |
| % Income <$20,000 | 29.0 (21.1-32.1) | 26.3 (20.3-28.8) | 23.2 (16.5-29.4) | 24.0 (18.5-31.7) | 32.0 (26.4-35.4) | **0.0012** |
| % Attained college education | 11.8 (9.7-12.7) | 11.4 (9.7-16.6) | 12.7 (10.2-16.6) | 13.1 (10.6-16.6) | 10.5 (9.4-12.4) | **0.3281** |
| % Adult obesity | 27.1 (22.8-29.9) | 26.0 (23.8-28.8) | 25.3 (22.5-27.7) | 24.8 (22.1-27.3) | 26.0 (24.5-29.4) | **0.0716** |
| % Adult smoking | 18.1 (17.6-20.0) | 18.2 (16.4-19.7) | 17.2 (15.7-18.4) | 18.0 (17.1-19.4) | 19.6 (18.0-21.4) | **<0.0001** |
| % Rural | 76.1 (67.7-100.0) | 65.6 (31.4-81.8) | 60.0 (28.1-79.4) | 56.5 (20.6-73.1) | 60.4 (46.9-80.9) | **0.1971** |
| ^a^Significance determined using Kruskal-Wallis tests, p values <0.05.   ^b^IQR = interquartile range.  ^c^ICU = intensive care unit, ICU bed tally does not include Veterans Affairs hospitals, which are sure to play a role in treating COVID-19 patients, because VA hospitals do not file cost reports to CMS.  ^d^PCP = primary care physicians | | | | | | |
