# Supplementary material for: Racial Disparities in the Epidemiology of COVID-19 in Georgia: Trends Since State-Wide Reopening
Source: Health Equity. 2021 Mar 2;5(1):91–9. doi: 10.1089/heq.2020.0089 (PMC7990566; doi:10.1089/heq.2020.0089)
Supplement: Supplemental data [file Supp_Table2.docx]

| **Supplemental Table 2. County Level Characteristics Comparisons by Coronavirus Disease 2019 (COVID-19) Incidence Rates, Among Georgia Counties March 3 through on June 30, 2020.** | | | | | |
| --- | --- | --- | --- | --- | --- |
|  | Quartiles of Incidence Rate | | | | |
| Characteristic | 1^st^ Quartile (116.4822-454.958)  (*N* = 39) | 2^nd^ Quartile (454.958-624.288)  (*N* = 40) | 3^rd^ Quartile (624.288-1003.57)  (*N* = 40) | 4^th^ Quartile (1003.57-4561.23)  (*N* = 40) | *p* value^a^ |
|  | Presented as Median (IQR)^b^ | | | |  |
| Race |  |  |  |  |  |
| % NH-White | 71.2 (57.6-85.9) | 70.9 (55.0-86.7) | 59.2 (50.4-73.3) | 55.6 (40.2-63.5) | <0.0001 |
| % NH-Black | 23.2 (9.5-33.7) | 24.7 (8.8-37.5) | 30.8 (17.4-38.9) | 32.1 (22.6-45.0) | 0.0235 |
| % Hispanic | 4.0 (2.8-7.7) | 5.1 (3.3-8.1) | 6.0 (4.0-10.2) | 5.0 (3.0-12.2) | 0.2276 |
| % Female Sex | 51.5 (49.0-55.3) | 52.2 (50.9-57.0) | 51.7 (48.7-54.1) | 49.1 (47.6-51.2) | 0.0003 |
| % Age 65+ | 17.6 (15.8-20.8) | 16.8 (15.6-18.7) | 17.6 (15.4-19.2) | 16.0 (14.8-17.9) | 0.1405 |
| ICU^c^ beds per 100,000 population | 0.0 (0.0-9.9) | 0.0 (0.0-19.2) | 3.0 (0.0-25.4) | 0.0 (0.0-30.2) | 0.3050 |
| PCP^d^ per 10,000 population | 3.7 (1.4-5.6) | 4.2 (2.8-6.1) | 4.1 (2.6-5.6) | 3.8 (1.5-6.2) | 0.5080 |
| % Uninsured | 12.4 (11.4-13.6) | 12.8 (12.1-14.4) | 13.4 (12.0-15.2) | 12.4 (10.8-15.2) | 0.2569 |
| % Income <$20,000 | 24.2 (17.5-29.4) | 26.5 (17.0-30.2) | 26.5 (21.6-32.5) | 31.9 (25.7-35.0) | 0.0012 |
| % Attained college education | 11.8 (9.5-14.9) | 12.2 (10.2-16.3) | 11.7 (10.3-14.9) | 10.7 (9.4-13.4) | 0.3281 |
| % Adult obesity | 26.5 (23.7-29.7) | 25.8 (22.9-27.7) | 24.2 (21.7-27.3) | 26.0 (24.6-28.5) | 0.0716 |
| % Adult smoking | 17.5 (16.1-19.2) | 17.8 (16.5-19.3) | 18.2 (17.1-19.2) | 19.8 (18.1-21.3) | <0.0001 |
| % Rural | 72.1 (56.6-100.0) | 66.3 (32.1-80.5) | 60.2 (38.4-80.6) | 58.9 (41.3-74.7) | 0.1971 |
| ^a^Significance determined using Kruskal-Wallis tests, p values <0.05.   ^b^IQR = interquartile range.  ^c^ICU = intensive care unit, ICU bed tally does not include Veterans Affairs hospitals, which are sure to play a role in treating COVID-19 patients, because VA hospitals do not file cost reports to CMS.  ^d^PCP = primary care physicians | | | | | |
