# Supplementary figures and images for: Racial Disparities in the Epidemiology of COVID-19 in Georgia: Trends Since State-Wide Reopening
Source: Health Equity. 2021 Mar 2;5(1):91–9. doi: 10.1089/heq.2020.0089 (PMC7990566; doi:10.1089/heq.2020.0089)

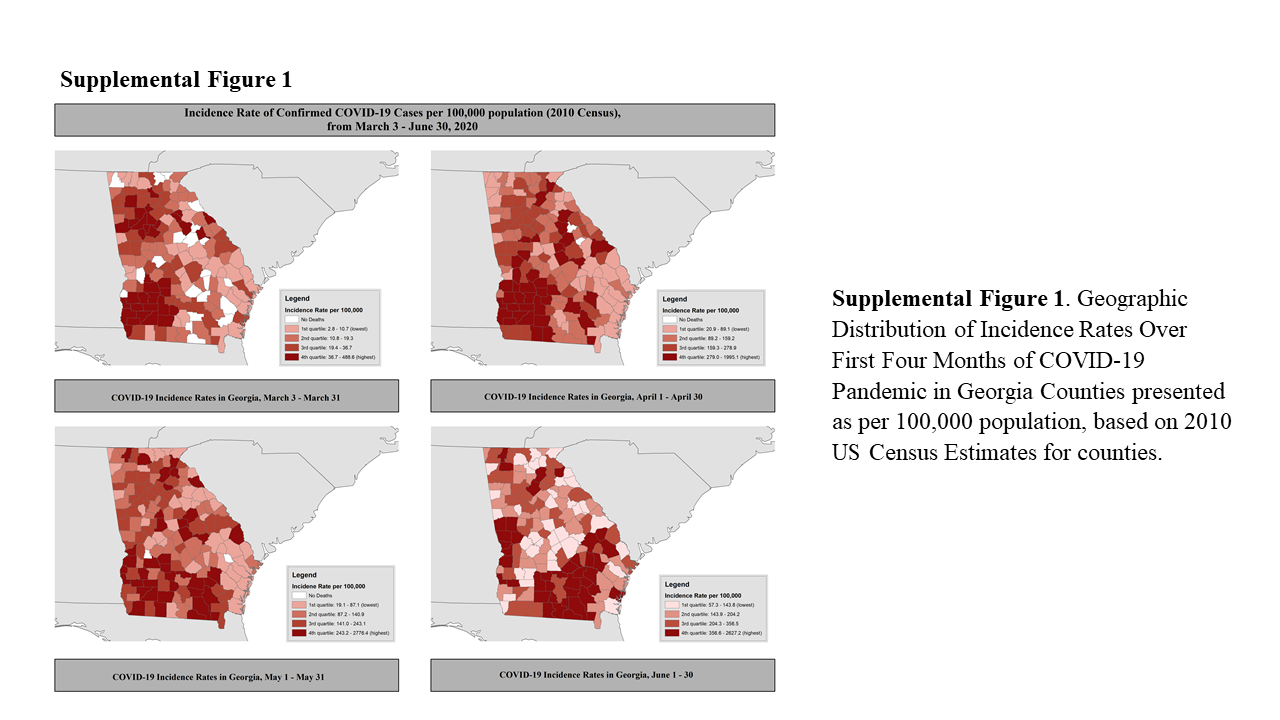

Supplement: Supplemental data [file Supp_Fig1.tif]

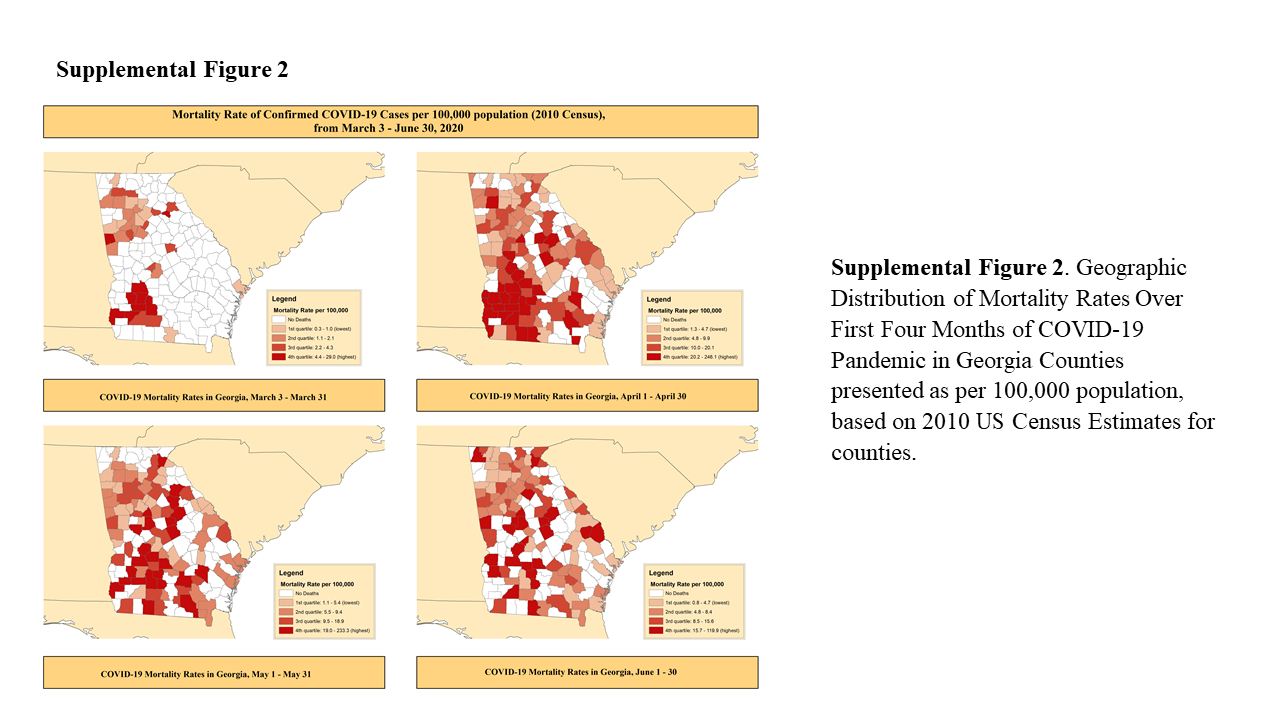

Supplement: Supplemental data [file Supp_Fig2.tif]

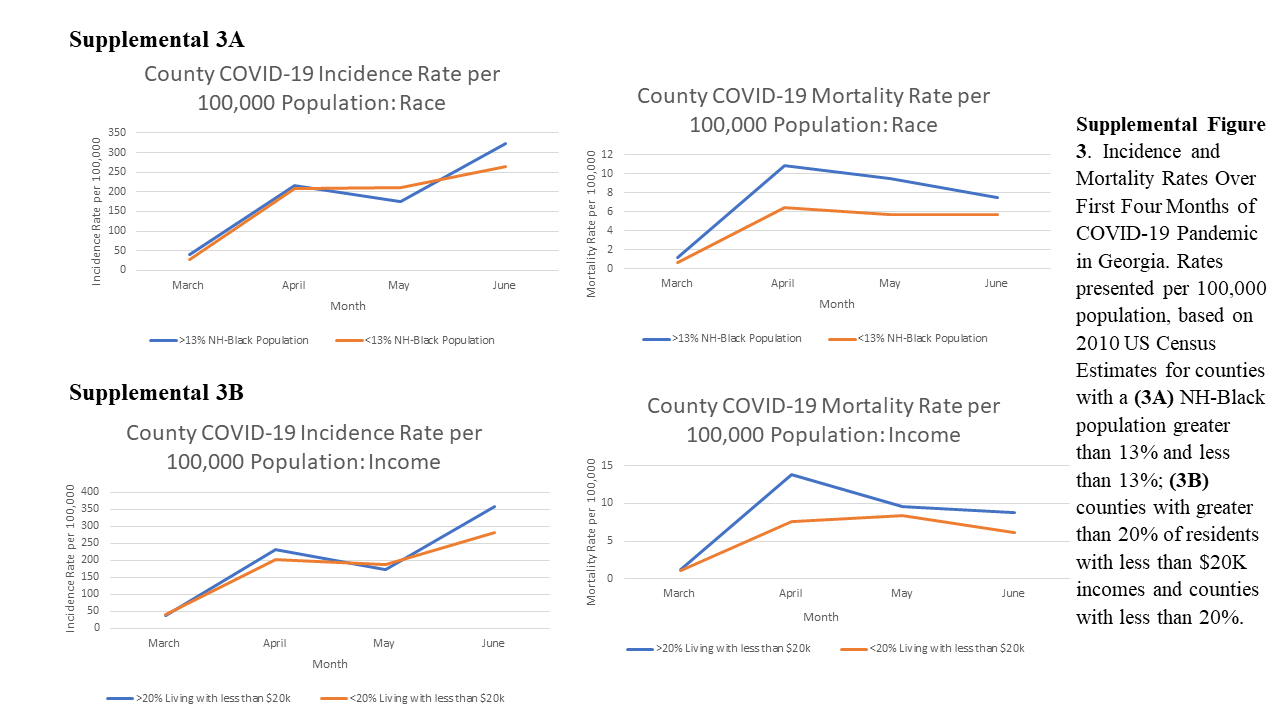

Supplement: Supplemental data [file Supp_Fig3.tif]
