# Supplementary material for: Racial Disparities in the Epidemiology of COVID-19 in Georgia: Trends Since State-Wide Reopening
Source: Health Equity. 2021 Mar 2;5(1):91–9. doi: 10.1089/heq.2020.0089 (PMC7990566; doi:10.1089/heq.2020.0089)
Supplement: Supplemental data [file Supp_Table3.docx]

| **Supplemental Table 3. The Association Between County Level Characteristics with Highest Quartile of Coronavirus Disease 2019 (COVID-19) Incidence Rates, Among Georgia Counties Since Reopening on April 24, 2020. Presented as Odds Ratios (ORs) and 95% Confidence Intervals (CIs), Estimated Using Binary Logistic Regression.** | | | | |
| --- | --- | --- | --- | --- |
| Characteristic | Unadjusted   OR^b^ (95% CI) | *p* value^a^ | Adjusted^c^  OR (95% CI) | *p* value^a^ |
| % NH-Black |  |  |  |  |
| 1^st^ quartile (0.7 – 14.7) | 1.00 (Referent) | 0.371 | 1.0 (Referent) | 0.735 |
| 2^nd^ quartile (14.8 – 28.0) | 2.09 (0.69-6.35) | 0.618 | 1.57 (0.40-6.16) | 0.281 |
| 3^rd^ quartile (28.1 – 38.7) | 1.83 (0.59-5.66) | 0.932 | 0.87 (0.23-3.24) | 0.620 |
| 4^th^ quartile (38.7 – 78.0) | 2.65 (0.89-7.90) | 0.194 | 0.84 (0.20-3.55) | 0.602 |
| % Hispanic |  |  |  |  |
| 1^st^ quartile (1.4-3.1) | 1.00 (Referent) | 0.827 | 1.00 (Referent) | 0.011 |
| 2^nd^ quartile (3.2-5.1) | 1.17 (0.35-3.84) | 0.259 | 1.44 (0.37-5.54) | 0.105 |
| 3^rd^ quartile (5.2-8.5) | 2.36 (0.78-7.09) | 0.352 | 4.85 (1.23-19.16) | 0.130 |
| 4^th^ quartile (8.6-36.4) | 3.30 (1.12-9.72) | 0.033 | 7.75 (1.85-32.49) | 0.008 |
| % Income <$20,000 |  |  |  |  |
| 1^st^ quartile (8.7-19.4) | 1.00 (Referent) | 0.014 | 1.00 (Referent) | 0.008 |
| 2^nd^ quartile (19.5-27.0) | 2.19 (0.60-7.96) | 0.603 | 4.51 (0.97-20.89) | 0.741 |
| 3^rd^ quartile (27.1-32.1) | 3.32 (0.96-11.53) | 0.462 | 8.44 (1.75-40.75) | 0.183 |
| 4^th^ quartile (32.2-47.8) | 6.47 (1.93-21.67) | 0.003 | 18.24 (3.40-97.82) | 0.002 |
| ^a^Significance determined using Wald Chi-Square from Logistic Regression.  ^b^Odds Ratio estimate the odds of being in the highest quartile for COVID-19 incidence rate.  ^c^Adjusted for age, obesity, and rurality | | | | |
